# Health and economic impact of dapagliflozin for type 2 diabetes patients who had or were at risk for atherosclerotic cardiovascular disease in the Italian general practitioners setting: a budget impact analysis

**DOI:** 10.1007/s00592-024-02276-3

**Published:** 2024-04-18

**Authors:** Paolo Angelo Cortesi, Ippazio Cosimo Antonazzo, Pasquale Palladino, Marco Gnesi, Silvia Mele, Marco D’Amelio, Elena Zanzottera Ferrari, Giampiero Mazzaglia, Lorenzo Giovanni Mantovani

**Affiliations:** 1 Research Centre on Public Health (CESP), University of Milano-Bicocca, Via Pergolesi 33, Monza, MB Italy; 2 Istituto Auxologico Italiano-IRCCS, Milan, Italy; 3 Cegedim Health Data, Milan, Italy; 4 Medical Evidence, Biopharmaceuticals Medical, AstraZeneca, Milan, Italy; 5 Value & Access, AstraZeneca, Milan, Italy

**Keywords:** Dapagliflozin, Italy, Budget impact analysis, General Practitioners

## Abstract

**Aim:**

In 2022, in Italy, general practitioners (GPs) have been allowed to prescribe SGLT2i in Type 2 Diabetes (T2D) under National Health Service (NHS) reimbursement. In the pivotal clinical trial named DECLARE-TIMI 58, dapagliflozin reduced the risk of hospitalization for heart failure, CV death and kidney disease progression compared to placebo in a population of T2D patients. This study evaluated the health and economic impact of dapagliflozin for T2D patients who had or were at risk for atherosclerotic cardiovascular disease in the Italian GPs setting.

**Methods:**

A budget impact model was developed to assess the health and economic impact of introducing dapagliflozin in GPs setting. The analysis was conducted by adopting the Italian NHS perspective and a 3-year time horizon. The model estimated and compared the health outcomes and direct medical costs associated with a scenario with dapagliflozin and other antidiabetic therapies available for GPs prescription (scenario B) and a scenario where only other antidiabetic therapies are available (scenario A). Rates of occurrence of cardiovascular and renal complications as well as adverse events were captured from DECLARE-TIMI 58 trial and the literature, while cost data were retrieved from the Italian tariff and the literature. One-way sensitivity analyses were conducted to test the impact of model parameters on the budget impact.

**Results:**

The model estimated around 442.000 patients eligible for the treatment with dapagliflozin in the GPs setting for each simulated year. The scenario B compared to scenario A was associated with a reduction in the occurrence of cardiovascular and renal complication (−1.83%) over the 3 years simulated. Furthermore, the scenario A allowed for an overall cost saving of 102,692,305€: 14,521,464€ in the first year, 33,007,064€ in the second and 55,163,777€ in the third. The cost of cost of drug acquisition, the probability of cardiovascular events and the percentage of patients potentially eligible to the treatment were the factor with largest impact on the results.

**Conclusions:**

The use of dapagliflozin in GPs setting reduce the number of CVD events, kidney disease progression and healthcare costs in Italy. These data should be considered to optimize the value produced for the T2D patients who had or were at risk for atherosclerotic cardiovascular disease.

**Supplementary Information:**

The online version contains supplementary material available at 10.1007/s00592-024-02276-3.

## Introduction

Type 2 diabetes (T2D) is a chronic metabolic disorder characterized by insulin resistance and eventual functional failure of pancreatic beta cells [1]. Over the last few decades, the prevalence of T2D has increased globally, rising from 3.9 to 6.0% in men and from 3.5 to 5.0% in women in the period 1990–2019 [2]. The worldwide incidence of the disease rises in parallel with age, peaking around 55–59 years [3]. Previous studies suggested a significant and ongoing increase in the prevalence and incidence of diabetes, projecting that by 2035, approximately 590 million (1 out of 10 person) will be affected by this disease [4]. Factors such as rapid economic development, which can be considered a proxy for changes in lifestyle habits (i.e., diet), have contributed to the rising burden of diabetes in many parts of the world [5].

This disease poses a substantial public health concern due to its impact on functional capacities and quality of life, leading to significant morbidity and premature mortality [6]. Diabetes also comes with a considerable economy burden, encompassing both direct and indirect costs associated with reduced productivity due to mortality and morbidity linked with the disease [7]. Complications such as neuropathy, renal disease, retinopathy, myocardial infarction and coronary artery disease are the major drivers of the costs associated with diabetes [7–10]. To this regard, diabetes is a leading cause of CVDs which, in turn, are leading cause of death and disability in these patients. In patients with diabetes, the risk of CVDs mortality has been estimated to be one–threefold higher in males and two–fivefold in females compared to individuals without diabetes [10–12].

Recent clinical trials of the glucose-lowering drug class of sodium–glucose co-transported-2 inhibitors (SGLT2is) have demonstrated benefits in preventing CVD events and kidney diseases progression in patients with T2D [13–17]. Findings from these trials showed a reduction in CV and renal outcomes in treated individuals compared to the standard of care (SoC) group [13–17]. The favorable benefit–risk profile of these treatments endorses the 2020 European Society of Cardiology guideline recommendations for the administration of SGLT2is as first-line therapy in patients with T2D and established CVD [18–20].

Dapagliflozin was the first drug from the SGLT2i class to reach the global market. In the pivotal randomized clinical trial DECLARE-TIMI 58, dapagliflozin reduced the risk of hospitalization for heart failure or CV death compared with placebo, as well as reducing kidney disease progression, in individuals who have or are at risk for atherosclerotic cardiovascular disease. These findings were also corroborated by several real-world studies [21–28]. To date, the DECLARE-TIMI 58 trial is the longest, largest and broadest SGLT2i Cardiovascular Outcome Trial (CVOT), including both a high-risk, secondary prevention group with established CVD disease (i.e., heart failure) and a large primary prevention group [29]. While the safety and clinical efficacy of dapagliflozin have been well described, the economic impact of this therapy in some countries (i.e., Italy) has been poorly investigated. Although previous studies have been conducted to assess its cost-effectiveness [29–34], evidence on its budget impact on the healthcare system is still scant. Further, in Italy, the possibility for general practitioners (GPs) to prescribe SGLT2i in T2D under National Health Service (NHS) reimbursement has been opened since 2022, under the recommendation issued by the Italian Medicine Agency (AIFA). Considering this new scenario, the aim of this study was to assess the health consequence and economic impact of dapagliflozin for T2D patients who had or were at risk for atherosclerotic cardiovascular disease in the Italian GPs setting.

## Methods

### Model overview

Following the guideline outlined by the International Society for Pharmacoeconomics and Outcomes Research (ISPOR) on “Principles of Good Practice for Budget Impact Analysis,” a budget impact analysis (BIA) was conducted utilizing a model constructed in Microsoft Excel [35]. This model aimed to assess the impact of introducing dapagliflozin in the primary care setting for the treatment of T2D patients who had or were at risk for atherosclerotic cardiovascular disease in comparison with a scenario where only other antidiabetic therapies (GLP1 receptor agonists, DPP4 inhibitors, sulfonylureas and metformin) were available for prescription by GPs. The model is based on epidemiological data concerning T2D patients meeting the eligibility criteria of the DECLARE-TIMI 58 trial and receiving treatment from GPs. Economic data input for the model encompassed the costs of hypoglycemic therapies, management of adverse events and handling cardiovascular and renal complications. Specifically, the model initially estimated the cost per patient within each treatment regimen, and this cost was then applied to the total number of patients treated with each therapeutic alternative included in the simulation, based on the entered market share. The model compared two scenarios: the scenario without dapagliflozin (scenario A) with GLP1 receptor agonists, DPP4 inhibitors, sulfonylureas and metformin available as treatment options, versus the counterfactual scenario in which dapagliflozin is available in the country within the other hypoglycemic therapies (Scenario B).

The analysis was conducted from the perspective of Italian NHS using a 3-year time horizon.

### Population

The model population was determined through a two-step processes. Initially, the number of Italian diabetes cases was computed by multiplying the T2D prevalence rate (6.20%) within the Italian population with the total number of inhabitants in the country [36]. This calculation incorporated population data from 2023 and accounted for the anticipated demographic changes over the subsequent two years, as obtained from the DemoISTAT dataset (https://demo.istat.it/) (Table 1) [37].

**Table 1:** Number of patients eligible to the treatment during the study period

	Year 1	Year 2	Year 3	References
Italian population	58,850,717	58,696,239	58,559,913	[37]
Prevalence of diabetes in Italy	6.20%	6.20%	6.20%	[36]
Number of patients with diabetes in Italy	3,648,744	3,639,167	3,630,715	
Prevalence of type 2 diabetes	87.50%	87.50%	87.50%	[40]
Percentage of patients currently in treatment with general practitioners (GPs)	50.00%	50.00%	50.00%	[41]
Number of patients currently treated with GPs	1,596,326	1,592,135	1,588,438	
Percentage of patients who meet the criteria of the DECLARE-TIMI 58 study	48.30%	48.30%	48.30%	[42]
Number of patients who meet the criteria of the DECLARE-TIMI 58 study	771,025	769,001	767,215	
Percentage of patients who use antidiabetic drugs among patients with T2D eligible for the DECLARE-TIMI 58 study	67.90%	67.90%	67.90%	[42]
Number of patients who use antidiabetic drugs among patients with T2D eligible for the DECLARE-TIMI 58 study	523,526	522,152	520,939	
Percentage of patients who use antidiabetic drugs other than insulin among patients with T2D eligible for the DECLARE-TIMI 58 study	84.70%	84.70%	84.70%	[42]
Number of patients who use antidiabetic drugs other than insulin among patients with T2D eligible for the DECLARE-TIMI 58 study	443,427	442,263	441,236	

Subsequently, as indicated in Table 1, the count of patients who met the eligibility criteria for enrollment in the DECLARE-TIMI 58 clinical trial treated by GPs was estimated by using The Health Improvement Network (THIN) database (for more details see “THIN methods description” in Supplementary Material). Briefly, the THIN database gathers fully anonymized electronic medical records collected from GPs that have joined the THIN® network. The Italian database collects longitudinal anonymized patient-level information on healthcare services reimbursed by the NHS, dispensed to around 1 million active patients with a mean of around 9 years of clinical data history and registered with 550 Italian GPs, distributed over the whole country. Several published reports have demonstrated the representativeness of the collected information in terms of patients' demographics, the prevalence of chronic conditions and mortality rates [38, 39]. This estimation was accomplished by multiplying the number of diabetes affected individuals in Italy, for each studied year, by various prevalence factors, which included the prevalence of T2D in the Italian population with diabetes (87.50%), the proportion of T2D subjects treated by general practitioners (GPs) (50.0%) and the percentage of patients meeting the eligibility criteria for participation in the DECLARE-TIMI 58 clinical trial (48.3%) (Table 1) [36, 40–42].

The target population was further defined by applying the prevalence of subjects undergoing treatment with antidiabetic medications (67.90%) and the prevalence of individuals not receiving insulin therapy (84.70%), estimated using the THIN database [42]. Consequently, the target population figures were determined to be 443,427 in the first year, 442,263 in the second year and 441,236 in the third years of the simulation (Table 1).

### Treatments

Recently, the Italian Medicine Agency (AIFA) issued a regulatory note outlining prescription guidelines for SGLT2 inhibitors in the GPs setting. The Italian Note serves as a tool to be employed by AIFA to ensure the appropriateness of medicines use, guide therapeutic choices toward effective and validated molecules, and regulate pharmaceutical expenditure. Specifically, NOTA 100 provides directives for GPs and NHS specialists authorized by the regions regarding the prescription of SGLT2i, GLP1 receptors agonists, DPP4 inhibitors and other hypoglycemic therapies [43].

Aligned with the directive of NOTA AIFA, the present study considered the following therapeutic options: SGLT2i (i.e., dapagliflozin, canagliflozin, empagliflozin, ertugliflozin), GLP1 receptor agonists (i.e., dulaglutide, exenatide LAR, liraglutide, lixisenatide, semaglutide oral and semaglutide subcutaneous), DPP4 inhibitors (i.e., alogliptin, linagliptin, saxagliptin, sitagliptin and vildagliptin), sulfonylureas (i.e., gliclazide and glimepiride) and metformin. We used dapagliflozin as SGLT2i treatment to be consistent with the epidemiological and clinical data retrieved using the DECLARE-TIMI 58 trial patients’ characteristics and treatment effects.

### Costs data

In accordance with the analysis perspective, the model incorporated only direct healthcare cost paid by the Italian NHS. These resources encompassed: drug acquisition costs; costs related to the management of cardiovascular and kidney complications; and costs associated with the management of adverse events.

More specifically, the drug acquisition costs for each therapy were computed as an annual expense by multiplying the unit cost by average dose administered over the course of 1 year of treatment. The dose and treatment regimen for each therapy were obtained from the European public assessment report (EPAR) provided by the European Medicine Agency and by RCP as documented on the AIFA website. The ex-factory price for package, net of statutory discount (−5%, followed by −5%) was considered, in compliance with legal requirements for each reimbursed drug. Table 2 shows the prices for each drug included in the analysis, obtained through reference to the Italian official gazettes or retrieved from the AIFA website—List of Drugs of class A and H [44]. For GLP1 receptor agonists, DPP4 inhibitors and sulfonylureas drug class, the model used an average price of the different products included in each class [45–53].

**Table 2:** Treatments, complications and adverse event occurrence costs

Treatment Costs					
	Cost per pack (ex-factory with statutory discount)	Number of posological units	Dose per unit	Cost per month of treatment*	References
SGLT2i					
*Dapagliflozin*	32.29 €	28	10 mg	35.10 €	[45]
GLP1ra					[46–49]
*Dulaglitide*	278.98€	12	3 mg	101.09 €	
*Exenatride LAR*	89.96 €	4	2 mg	97.79 €	
*Liraglutide*	68.00 €	2	20 mcg	81.04 €	
*Semaglutide oral*	119.81 €	30	14 mg	121.55 €	
DPP4i					[44, 50–52]
*Alogliptin*	33.85 €	28	25 mg	36.80 €	
*Linagliptin*	37.51 €	28	5 mg	40.78 €	
*Saxagliptin*	35.63 €	28	5 mg	38.73 €	
*Sitagliptin*	15.78 €	28	100 mg	17.16 €	
*Vildagliptin*	17.36 €	56	50 mg	18.88 €	
Sulfonylureas					[44]
*Gliclazide*	2.86 €	30	60 mg	5.81 €	
*Glimepiride*	2.72 €	30	4 mg	2.76 €	
Metformin*	3.39 €	60	500 mg	6.89 €	[53]

Costs associated with the management of cardiovascular and renal complications					
	Annual costs				References
Cardiovascular complications					
*Hospitalization for heart failure*	4,898 €				[54]
*Myocardial infarction*	11,137 €				[55]
*Ischemic stroke*	10,058 €				[56]
Renal complications					
*Decrease in eGFR by 40%*	4,508 €				[57]
*ESRD*	36,800 €				[58]

Costs associated with adverse event occurrence					
Major hypoglycemic event	685 €				[61]
Diabetic ketoacidosis	2,529 €				[62]
Amputation	7,698 €				[63]
Genital infection	2,701 €				[63]
Gastrointestinal event	3,484 €				[63]

^*^The monthly cost was estimated by considering the dosage included into the recommended Summary of Product or the average dosage used for the therapy

In the model, the costs related to the management of cardiovascular and renal complications were computed by multiplying the costs of healthcare resources used to manage the specified events by the annual occurrence of each event for each line of treatment [54–58]. Specifically, the likelihood of cardiovascular events and kidney complications (i.e., heart failure, myocardial infarction and ischemic stroke, decrease in eGFR by 40% and end-stage renal disease) were estimated as follows:

(1) For dapagliflozin, events rate from the treatment arm of the DECLARE-TIMI 58 clinical [16];

(2) For GLP1ra, applying to dapagliflozin rates the odds ratios estimates from a recent meta-analysis comparing the efficacy of GLP1ra vs SGLT2i on cardiovascular events and kidney complications [59];

(3) for DPP4i class, sulfonylureas and metformin, data from the placebo arm of the DECLARE-TIMI 58 trial, considering their high prevalence of use in the placebo arm of the aforementioned trial [16] and the no efficacy showed in reducing cardiovascular events and kidney complications [16] (Table 3).

**Table 3:** Probability of cardiovascular and renal complications for the drugs included in the model

	DPP4, Sulfonylureas e Metformin [16]		Dapagliflozin [16]		GLP1 [59]	
	Rate per 1000 pt year	Prob. yearly	Rate per 1000 pt year	Prob. yearly	OR** (95% CI)	Prob. yearly
Cardiovascular outcomes						
*Hospitalization for heart failure*	8.5	0.0084	6.2	0.0062	1.34 (1.17–1.54)	0.0083
*Myocardial infarction*	13.2	0.0131	11.7	0.0116	1.05 (0.93–1.19)	0.0122
*Ischemic stroke*	6.8	0.0067	6.9	0.0069	0.83 (0.71–0.98)	0.0057
Renal outcomes						
Overall*	7.00	0.0069	3.7	0.0037	–	–
*Decrease in eGFR by 40%*	5.95	0.0059	3.145	0.0031	1.10 (0.83.1.45)	0.0035
*ESRD*	0.98	0.0009	0.518	0.0005	1.10 (0.83.1.45)	0.0005

^*^85% Decrease in eGFR by 40 and 14% ESRD [60]

^**^ORs assessed comparing GLP1 versus SGLT2

For renal complications rates, the DECLARE-TIMI 58 trial [16] reported a composite outcome, encompassing a 40% reduction in the estimated glomerular filtration rate (eGFR), end-stage renal disease (ESRD) and death due to renal causes; to estimate the single event rate, these data were adjusted by utilizing the annual probability of each event as reported in a previous study [60]. The costs associated with the management of the CV events and kidney complications were based on data reported in the literature (Table 2) [54–58].

The model included also the cost associated with the following serious adverse events: major hypoglycemic events, severe gastrointestinal events, diabetic ketoacidosis, amputations and genital infections. The annual costs associated with their management was computed by multiplying the number of events in each year of the simulation, for each study treatment, by the cost of healthcare resources used (Supplementary Table 1) [61–63]. The annual probability of adverse drug reactions (ADRs) occurrence for dapagliflozin was based on data reported in the DECLARE-TIMI 58 trial: The annual probability was based on the percentage of ADR occurrence and the duration of follow-up in the treated group [16]. Similarly, annual probability of ADR for DPP4i, sulfonylureas and metformin was based on data from the placebo arm in the DECLARE-TIMI 58 trial [16]. The annual probability of ADR in the GLP1ra group was based on data reported in a recent meta-analysis [59] (Supplementary Table 1).

### Market shares

The market shares for all drugs included in the scenario A, without dapagliflozin, were retrieved from a recent market research study conducted by using the THIN database. The availability of dapagliflozin, used as proxy of SGLT2i, was assumed to lead to a redistribution of the market shares of other hypoglycemic therapies, which was captured in the scenario B (Supplementary Table 2). The variation in market shares was estimated through reasonable forecasts based on the market shares present in scenario A of the BIA (Supplementary Table 2). Specifically, in the model, over the 3-year time frame, the conditional probability of receiving dapagliflozin increased from 13.5% in the first year to 19.5% in the third year, resulting in a reduction in market shares for other antidiabetics (Supplementary Table 2).

### Statistical analysis

Through a comparison of the two scenarios simulated by the model, the health and economic consequences of introducing dapagliflozin were estimated, assessing the number of CV events and kidney progression and the costs associated with managing complications, adverse events and treatment. The outcomes were expressed as number of each CV events and kidney outcomes included in the model and as Euros (€) per year and presented both in aggregate and categorized by the respective cost components and treatment.

To assess the robustness of our study, a one-way sensitivity analysis was conducted. Specifically, the impact of each parameter included in the model was assessed. An iterative replacement approach of individual model input, by using their ± 10% change while holding other inputs constants, was performed. Subsequently, the resulting set of sensitivity analyses was ranked by the absolute magnitude of deviation from the base case to assess which input parameter most significantly affected the results.

## Results

### Population

**Fig. 1:**
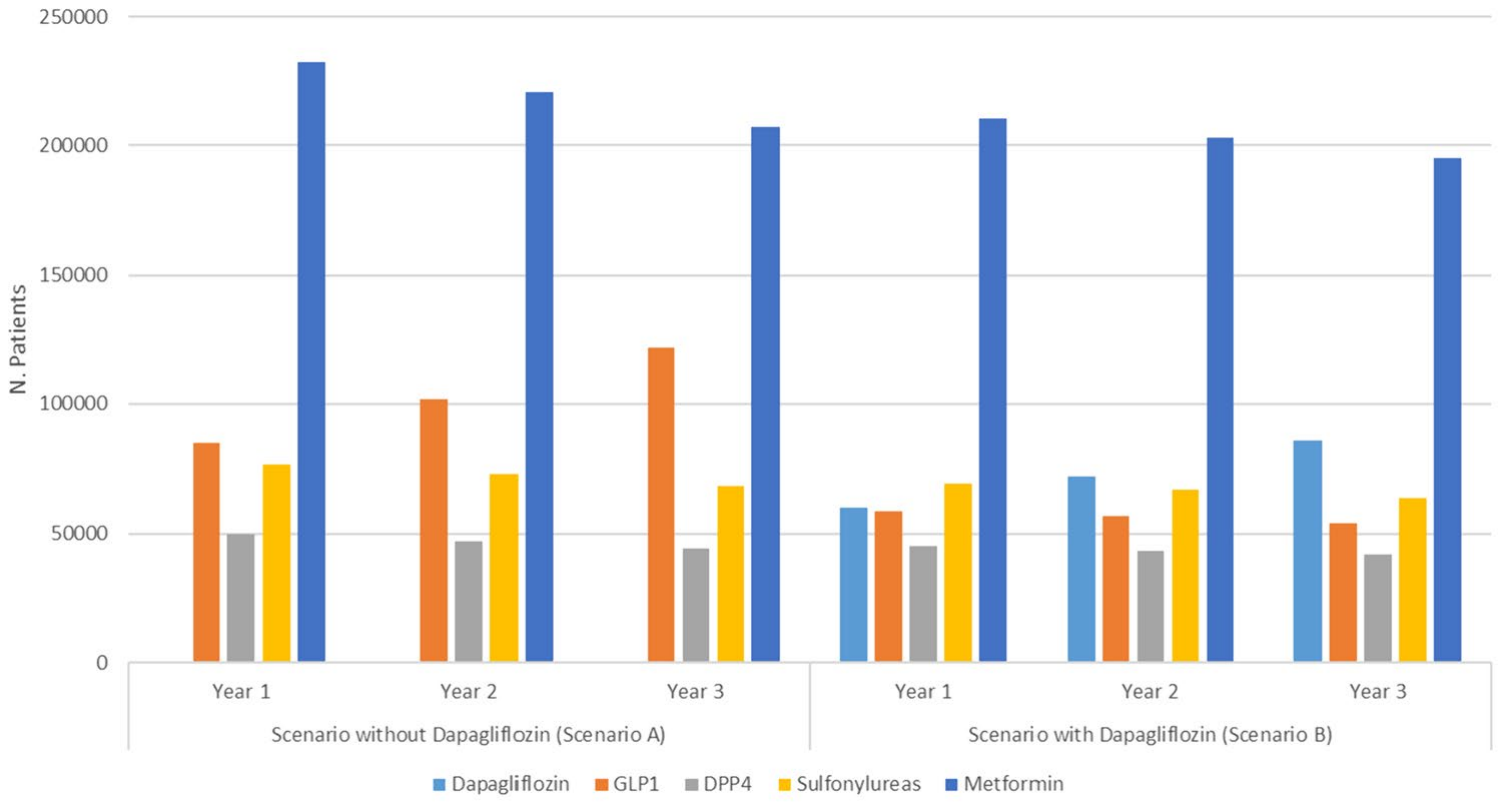
Number of treated individuals with each therapy within the scenario without dapagliflozin (Scenario A) and the scenario with dapagliflozin (scenario B)

Figure 1 reports the number of individuals diagnosed with T2D potentially eligible for the study treatment over the 3-year simulation period. Specifically, the estimated number of patients during the 3-year time frame was 443,427, 442,263 and 441,236 in the first, second and third year, respectively. As depicted in Fig. 1, in the scenario B (with dapagliflozin) the number of treated subjects increased from 60,081 in the first year to 86,090 in the third year. Furthermore, since an increase in the use of GLP1ra was simulated in the scenario without dapagliflozin, the availability of dapagliflozin resulted in a more pronounced reduction in the number of individuals treated with GLP1ra during the second and third years.

### Health/clinical impact

The clinical complications commonly observed in patients with diabetes, as included in the model (i.e., HF, myocardial infarctions, reduction of eGFR and ESRD), were averted when dapagliflozin was used instead of other hypoglycemic therapies (Table 4). The utilization of dapagliflozin resulted in an overall reduction of cardiovascular and renal complications by over 800 cases over the 3-year period, corresponding to a decrease of −1.82% in the occurrence of cardiovascular complication compared to the scenario without dapagliflozin. Specifically, the use of dapagliflozin was associated with a reduction of −4.43% in hospitalization for HF, −1.17% in myocardial infarction events, −3.89% in eGFR by 40% and −3.89% in ESRD. Conversely, an increase of 1.94% in the occurrence of ischemic stroke was observed (Table 4).

**Table 4:** Health impact of introducing dapagliflozin in the Italian market

Number of event	Year 1	Year 2	Year 3	Overall
Scenario without dapagliflozin				
Hospitalization for HF	3,738	3,725	3,713	11,176
Myocardial infarctions	5,738	5,708	5,677	17,123
Ischemic stroke	2,914	2,888	2,860	8,662
eGFR by 40%	2,420	2,372	2,316	7,108
ESRD	400	392	382	1,174
Total	15,210	15,085	14,948	45,243

Scenario with dapagliflozin				
hospitalization for HF	3,605	3,569	3,528	10,702
myocardial infarctions	5,673	5,642	5,610	16,925
ischemic stroke	2,948	2,944	2,941	8,833
eGFR by 40%	2,317	2,282	2,243	6,842
ESRD	383	3,77	370	1,130
Total	14,814	14,692	14,814	44,432

Change between the scenario with dapagliflozin and the scenario without dapagliflozin				
hospitalization for HF	− 133	− 156	− 185	− 474
myocardial infarctions	− 65	− 66	− 67	− 198
ischemic stroke	34	56	81	171
eGFR by 40%	− 103	− 90	− 73	− 266
ESRD	− 17	− 15	− 12	− 44
Number of total event	− 284	− 271	− 256	− 811
Total change (%)	− 1.9	− 1.8	− 1.7	− 1.8

### Budget impact

The use of dapagliflozin yielded an overall budget reduction exceeding €102 million over the 3-year period, corresponding to a percentage decrease of −11.9% compared to the budget impact in the scenario without dapagliflozin. The availability of dapagliflozin resulted in a cost saving of €14,521,464, €33,007,074 and €55,163,777 during the first, second and third years of simulation, respectively (Table 5). The primary drivers of the budget impact were drug costs, followed by the management of adverse event and cardiovascular/renal complications. Detailed annual costs for each treatment in the two scenarios are provided in Supplementary Table 3.

**Table 5:** Budget impact result scenario with dapagliflozin (scenario B) versus scenario without dapagliflozin (scenario A)

	Year 1	Year 2	Year 3	Overall
Scenario without dapagliflozin				
Overall	€ 301,677,535	€ 320,321,896	€ 342,847,243	€ 964,846,674
*Treatments*	€ 143,572,673	€ 161,635,040	€ 183,335,321	€ 488,543,034
*Complications management*	€ 137,141,265	€ 135,969,663	€ 134,682,185	€ 407,793,113
*Adverse event management*	€ 20,963,597	€ 22,717,193	€ 24,829,738	€ 68,510,528

Scenario with dapagliflozin				
Overall	€ 287,156,071	€ 287,314,832	€ 287,683,466	€ 862,154,369
*Treatments*	€ 133,504,833	€ 134,759,839	€ 136,315,417	€ 404,580,089
*Complications management*	€ 135,013,030	€ 134,074,985	€ 133,067,317	€ 402,155,332
*Adverse event management*	€ 18,638,208	€ 18,480,008	€ 18,300,732	€ 55,418,949

Change between the scenario with dapagliflozin and the scenario without dapagliflozin				
Overall	−€ 14,521,464	−€ 33,007,064	−€ 55,163,777	−€ 102,692,305
*Treatments*	−€ 10,067,840	−€ 26,875,201	−€ 47,019,904	−€ 83,962,945
*Complications management*	−€ 2,128,235	−€ 1,894,678	−€ 1,614,868	−€ 5,637,781
*Adverse event management*	−€ 2,325,389	−€ 4,237,185	−€ 6,529,006	−€ 13,091,579
Total change (%)	− 5.06	− 11.49	− 19.18	− 11.91

**Fig. 2:**
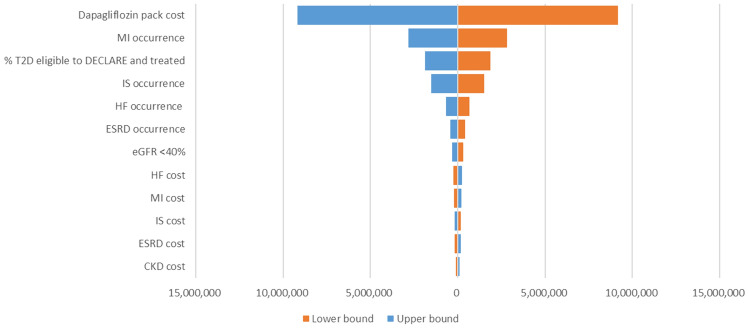
One-way sensitivity analysis. % = percentage; MI = myocardial infarction; IS = ischemic stroke; HF = heart failure; ESRD = end-stage renal disease; CKD = chronic kidney disease

One-way deterministic sensitivity analysis results are reported in Fig. 2. The cost of drug acquisition, the probability of cardiovascular events (i.e., myocardial infarction and ischemic stroke) and the percentage of patients potentially eligible to the treatment were the factors with largest impact on the results.

## Discussion

The study, for the first time, attempted to analyze the affordability of dapagliflozin compared to other hypoglycemic therapies, adopting the perspective of the Italian NHS and the GP setting. The findings suggested that the use of dapagliflozin resulted in an overall budget saving of €102,692,305 and a reduction in the occurrence of cardiovascular events and kidney diseases progression over 3 years.

The cost savings associated with dapagliflozin increased during the observed period, from €14 million in the first year to €55 million in the last. The positive impact of dapagliflozin use was primarily attributed to reductions in drug cost. Notably, the most significant impact on treatment costs was observed against the GLP1ra treatment. This could be attributed to the model’s assumption that in the absence of dapagliflozin, the GLP1ra could show an increased use over the time horizon. This is because GLP1ra as well as SGLT2is demonstrated efficacy in improving cardiovascular and renal outcome in patients with T2D with or at high risk for cardiovascular disease [64]. Furthermore, dapagliflozin is the only SGLT2is that showed a significant risk reduction in all-cause mortality in CKD and heart failure by 31 and 17%, respectively [65, 66]. Consequently, the availability of dapagliflozin could lead to a switch from patients treated with GLP1ra, especially during the second and third years due to the improvement in clinical and economic outcomes.

Regarding cardiovascular and renal complications, the scenario with dapagliflozin resulted in a reduction of −1.85% in the occurrence of these events with a more pronounced reduction for hospitalization for HF, eGFR by 40% and ESRD. These results were in line with those reported in the pivotal clinical trial [16], where dapagliflozin demonstrated a reduction in the risk of cardiovascular events, hospitalization for heart failure and progression of renal disease in diabetic patients, both with and without established cardiovascular disease [16].

Previous health economic evaluations indicated that dapagliflozin was a cost-effective treatment compared with other hypoglycemic options [67, 68]. This was particularly evident in high-risk patients with T2D, where dapagliflozin was associated with a reduction in the occurrence of cardiovascular events compared with other treatments [67]. Similar positive results were reported by Schoonhoven and colleagues, demonstrating cost-effectiveness of SGLT2i in T2D [68]. To the best of our knowledge this is the first study conducted to assess the budget impact of dapagliflozin compared with other hypoglycemic therapies in a European setting. Prior BIA involving another SGLT2i, empagliflozin, has indicated a cost-saving profile reporting a reduction of costs and CV events compare to standard of care [69–72]. It is crucial to note that this study mainly compares SGLT2i with a standard of care without specifically categorizing the alternative treatment within the main drug class, including GLP1ra, as done in our analysis, and only one was performed from Italian NHS perspective and limited prior to the “Nota 100” introduction.

In our study, we demonstrated that the use of dapagliflozin in the setting of primary care could lead to a budget reduction while improving the clinical outcome. Considering that dapagliflozin can be used for patients who had or were at risk for atherosclerotic cardiovascular disease, it represents an opportunity for the healthcare system. Given the high costs associated with treating people with comorbidities, the value of delaying disease progression and the development of HF and CKD offer a significant opportunity to avoid the cascade of costs associated with treating these comorbidities. Notably, a recent analysis using synthesized evidence from CVOTs and observational data demonstrated that SGLT2i, as a class, was cost-effective and often cost saving when compared to other therapies [29, 67]. The present economic analysis utilized data from DECLARE-TIMI 58 in isolation, considering the full spectrum of patients with the potential to benefit from dapagliflozin treatment.

This study has strengths and limitation. The main strength is that this is the first health and budget impact analysis of dapagliflozin compared with other hypoglycemic therapies, including GLP1ra drug class. Secondly, the study estimates direct medical costs of hypoglycemic therapies, CV events and management, as well as the budget impact of dapagliflozin as monotherapy in T2D patients with and without established cardiovascular events. Third, the study used data from DECLARE-TIMI 58 trial, that is to date the longest, largest and broadest SGLT2i CVOT, including both a high-risk, secondary prevention group with established CVD disease and a large primary prevention group [29]. Finally, the study benefits from data retrieved from an analysis conducted by THIN database, offering insights into the number of patients under the care of GPs and characteristics of patients, along with treatment pattern that can be considered as representative of Italian GPs setting.

As with all modeling analysis, this analysis has limitations which should be considered when interpreting the results. First, given that the BIA relied on the projected utilization rate of dapagliflozin, as indicated by the scenario analyses, the findings may not be generalizable to population with different adoption rates of the drug. Additionally, the analysis used wholesale acquisition costs (WAC) for drug acquisition without accounting for undisclosed discount, which are typically not publicly available and can vary substantially. To address these limitations, scenario analyses were conducted by varying the projected utilization rates of dapagliflozin and the drug acquisition cost for dapagliflozin. The findings corroborated those obtained in the main analysis. In this regard, in the analysis we used the ex-factory price following the recommendation by AIFA guideline for economic evaluation [73]. However, it should be noted that drug prices may have a confidential discount negotiated at national level between AIFA and the pharmaceutical company which must be applied to any regional or local purchase. This might result in potential lower price due to undisclosed discounts or tenders. Therefore, it is reasonable to speculate that if an additional discount were applied to the analysis the positive economic impact of the drugs might increase. In addition, in the model we included only dapagliflozin as a proxy of the entire SGLT2i class. Therefore, the findings may not be generalizable to other SGLT2is. In this context, it should be noted that, although with differences, all the SGLT2is showed a positive benefit–risk profile highlighting a beneficial effect as a class. Nevertheless, the positive effect of SGLT2is, especially for dapagliflozin [65, 66], on different endpoints was observed in T2D patients but also in other population such as patients with HF or CKD [17, 74–76] where dapagliflozin is the only SGLT2is that showed a significant risk reduction in all-cause mortality in patients with CKD and heart failure by 31% and 17%, respectively [65, 66]. In this study, we used dapagliflozin as SGLT2i treatment to be in line with the epidemiological and clinical data retrieved using the DECLARE-TIMI 58 trial patients’ characteristics and treatment effects. Based on the use of patients’ eligible criteria from DECLARE-TIMI 58 trial that are different to other CVOTs, the utilization of dapagliflozin is more appropriate than generalizing its application to the entire class of SGLT2i. Additionally, considering the cardiovascular and renal outcomes from the DECLARE-TIMI 58 trial, which is the longest, largest and most comprehensive CVOT for SGLT2i to date, further strengthens the appropriateness of dapagliflozin usage. In addition, the absence in the literature, of direct and reliable indirect comparison of different SGLT2i drugs in terms of efficacy and safety made difficult to make an analysis including all of them. Future studies should be performed to generate indirect comparison between SGLT2is in terms of efficacy and safety to incorporate these findings into new health economic models. Finally, in the model the clinical impact of hypoglycemic therapies, especially GLP1ra and SGLT2is, on BMI and related economic value was not considered. However, it should be noted that national and international guidelines recommend the use of different hypoglycemic therapies according with the disease severity regardless the weight of the patient [18–20, 43]. In this context, future studies should be conducted to evaluate the economic impact of GLP1ra and SGLT2is against each other and other hypoglycemic therapies in T2D patients considering also their effect on BMI [77, 78]. These types of studies might generate new evidence useful for clinicians and stakeholders to personalize the hypoglycemic therapies according with the patients’ characteristics.

## Conclusion

The study findings reveal that the availability of dapagliflozin in the GP setting in Italy could result in reduced total costs and a reduction in the CV and renal complications in patients with T2D who had or were at risk for atherosclerotic cardiovascular disease. The adoption of dapagliflozin in primary care as a consequence of the GPs opening can lead to an improvement from the clinical and economic point of view, and these data should help healthcare decision makers to optimize the value produced for the treatment and management of T2D patients who had or were at risk for atherosclerotic cardiovascular disease.

### Supplementary Information

Below is the link to the electronic supplementary material.

Supplementary file1 (DOCX 45 KB)

## Data Availability

The budget impact model structure and the data sources analyzed during the current study are available from the corresponding author on reasonable request. All data used to construct the model from both primary and secondary sources have also been presented within this manuscript.
