# Supplementary material for: Health and economic impact of dapagliflozin for type 2 diabetes patients who had or were at risk for atherosclerotic cardiovascular disease in the Italian general practitioners setting: a budget impact analysis
Source: Acta Diabetol. 2024 Apr 18;61(8):1017–28. doi: 10.1007/s00592-024-02276-3 (PMC11329540; doi:10.1007/s00592-024-02276-3)
Supplement: Supplementary file 1 — Supplementary file1 (DOCX 45 KB) [file 592_2024_2276_MOESM1_ESM.docx]

**Title:** Health and economic impact of dapagliflozin for type 2 diabetes patients who had or were at risk for atherosclerotic cardiovascular disease in the Italian general practitioners setting: a budget impact analysis.

**Authors:** Paolo Angelo Cortesi 1,2, Ippazio Cosimo Antonazzo 1,2, Pasquale Palladino 3, Marco Gnesi 4, Silvia Mele 5, Marco D’Amelio 5, Elena Zanzottera Ferrari 3, Giampiero Mazzaglia 1, Lorenzo Giovanni Mantovani 1,2.

**Affiliation:**

1. Research Centre on Public Health (CESP), University of Milano-Bicocca, Monza, Italy.

2. Istituto Auxologico Italiano-IRCCS, Milan, Italy.

3. Cegedim Health data, Milano, Italy.

4. Medical Evidence, Biopharmaceuticals Medical, AstraZeneca, Milan, Italy

5. Value & Access, AstraZeneca, Milan, Italy

**Corresponding author**

Ippazio Cosimo Antonazzo, PhD

Centre on Public Health (CESP), University of Milan-Bicocca,

Via Pergolesi 33, Monza (MB), Italy.

ippazio.antonazzo@unimib.it

**Supplementary material**

**THIN methods description**

The information gathered in the THIN database encompassed for demographic and clinical characteristics, treatments, lifestyle information, vital signs and laboratory test results. To reach the aim of the study, the number of patients managed by GPs who met the eligibility criteria for the DECLARE-TIMI 58 trial was estimated. Specifically, the following inclusion and exclusion criteria were applied to patient with T2D:

***Inclusion criteria***

- Age ≥ 40 years old

AND

- HbA1c ≥ 6.5% and < 12%

AND

- High risk of CV event, defined as
  - Pre-existing CVD

OR

- - Multiple CV risk factors, defined as:
    - Age ≥ 55 years old (males) or ≥ 60 years old (females)

AND

- - - At least one of dyslipidemia, hypertension, tobacco smoking

***Exclusion criteria***

The presence of **at least one** of the following criteria will make patients unable to comply with the eligibility criteria.

- AST or ALT > 3x upper limit normal
- Bilirubin > 2.5x upper limit normal
- Chronic kidney failure: eGFR < 60ml/min OR CKD stage 3-5 (including dyalisis and transplant)
- Ematuria
- Chronic cystitis or recurrent UTI
- Cancer
- Acute CV event in the two months before index date (defined as ACS, TIA, stroke, any revascularization, decompensated HF, sustained ventricular tachycardia)
- Current or recent treatment with pioglitazone (ATC: A10BG03).
- Current use of chronic (>30 consecutive days) treatment with an oral steroid at a dose equivalent to oral prednisolone ≥10 mg (e.g., betamethasone ≥1.2 mg, dexamethasone ≥1.5 mg, hydrocortisone ≥40 mg) per day.

To estimate the target population a multi-step approach was applied. Specifically, the number of patients eligible to DECLARE trial was estimated by dividing the number of patient meeting inclusion and exclusion criteria for DECLARE-TIMI 58 trial by the total number of T2D patients. Then, the number of patients with at least 1 antidiabetic treatment among those eligible to the DECLARE-TIMI trial was estimated. Specifically, estimate was performed by dividing the number of patients with at least 1 antidiabetic treatment and who met the eligibility for the DECLARE-TIMI trial by those who met the eligibility of the DECLARE-TIMI trial. Finally, the target population was estimated by dividing the number of patients who use antidiabetic treatments other than insulin among those who were eligible to the DECLARE-TIMI 58 trial and treated with at least 1 antidiabetic therapy by the number of patients eligible to the trial and treated with antidiabetic treatment.

Supplementary Table 1. Probability of ADR occurrence for each study treatment.

| Adverse event | DPP4, Sulfonylureas e Metformin [1] | Dapagliflozin [1] | GLP1 [2] | Cost per event | Reference |
| --- | --- | --- | --- | --- | --- |
| Major hypoglycemia | 0.24% | 0.17% | 0.48% | 685 € | [3] |
| Diabetic ketoacidosis | 0.02% | 0.07% | 0.02% | 2,529 € | [4] |
| Amputation | 0.31% | 0.34% | 0.35% | 7,698 € | [5] |
| Genital infection | 0.02% | 0.22% | 1.06% | 2,701 € | [5] |
| Severe gastrointestinal events | 0.00% | 0.00% | 2.13% | 3,484 € | [5] |

**Reference**

1. Wiviott SD, Raz I, Bonaca MP, et al (2019) Dapagliflozin and Cardiovascular Outcomes in Type 2 Diabetes. N Engl J Med 380:347–357. https://doi.org/10.1056/NEJMoa1812389

2. Palmer SC, Tendal B, Mustafa RA, et al (2021) Sodium-glucose cotransporter protein-2 (SGLT-2) inhibitors and glucagon-like peptide-1 (GLP-1) receptor agonists for type 2 diabetes: systematic review and network meta-analysis of randomised controlled trials. BMJ 372:m4573. https://doi.org/10.1136/bmj.m4573

3. Parekh W, Streeton SE, Baker-Knight J, et al (2018) The Economic Burden of Insulin-Related Hypoglycemia in Adults with Diabetes: An Analysis from the Perspective of the Italian Healthcare System. Diabetes Ther 9:1037–1047. https://doi.org/10.1007/s13300-018-0418-0

4. Haldrup S, Lapolla A, Gundgaard J, Wolden ML (2020) Cost-effectiveness of switching to insulin degludec from other basal insulins in real-world clinical practice in Italy. J Med Econ 23:271–279. https://doi.org/10.1080/13696998.2019.1669613

5. Ministero della Salute Gazzetta Ufficiale 2013 - Supplemento ordinario alla “Gazzetta Ufficiale„ n. 23 del 28 gennaio 2013 - Serie generale. DECRETO 18 ottobre 2012. Remunerazione delle prestazioni di assistenza ospedaliera per acuti, assistenza ospedaliera di riabilitazione e di lungodegenza post acuzie e di assistenza specialistica ambulatoriale

Supplementary Table 2. Market shares in the scenario without dapagliflozin (scenario A) compared with the scenario with dapagliflozin (scenario B).

|  | **Scenario without Dapagliflozin (Scenario A)** | | | **Scenario with Dapagliflozin (Scenario B)** | | |
| --- | --- | --- | --- | --- | --- | --- |
|  | **Year 1** | **Year 2** | **Year 3** | **Year 1** | **Year 2** | **Year 3** |
| **SGLT2** |  |  |  |  |  |  |
| Dapagliflozin | 0.0% | 0.0% | 0.0% | 13.5% | 16.3% | 19.5% |
| **GLP1** | 19.1% | 23.0% | 27.6% | 13.3% | 12.8% | 12.3% |
| **SoC** | 80.9% | 77.0% | 72.4% | 73.2% | 70.9% | 68.2% |
| **DPP4** | 11.2% | 10.7% | 10.0% | 10.2% | 9.8% | 9.5% |
| **Sulfonylureas** | 17.2% | 16.4% | 15.5% | 15.6% | 15.1% | 14.5% |
| **Metformin** | 52.4% | 49.9% | 46.9% | 47.4% | 45.9% | 44.2% |

Supplementary Table 3. Detailed budget impact results scenario with dapagliflozin (scenario B) Vs scenario without dapagliflozin (Scenario A).

|  | **Scenario without Dapagliflozin (Scenario A)** | | | **Scenario with Dapagliflozin (Scenario B)** | | |
| --- | --- | --- | --- | --- | --- | --- |
|  | **Year 1** | **Year 2** | **Year 3** | **Year 1** | **Year 2** | **Year 3** |
| **SGLT2 - Dapagliflozin** | | | | | | |
| Overall cost | € 0 | € 0 | € 0 | € 43,137,317 | € 51,628,902 | € 61,810,788 |
| Pharmacological therapy cost | € 0 | € 0 | € 0 | € 25,308,104 | € 30,290,007 | € 36,263,588 |
| Complications management cost | € 0 | € 0 | € 0 | € 15,752,859 | € 18,853,811 | € 22,572,026 |
| Adverse event management cost | € 0 | € 0 | € 0 | € 2,076,353 | € 2,485,084 | € 2,975,174 |
| **GLP1** | | | | | | |
| Overall cost | € 136,563,620 | € 163,446,183 | € 195,679,882 | € 94,544,045 | € 91,254,613 | € 87,318,388 |
| Pharmacological therapy cost | € 102,251,965 | € 122,380,275 | € 146,515,246 | € 70,789,822 | € 68,326,861 | € 65,379,614 |
| Complications management cost | € 22,967,024 | € 27,488,085 | € 32,909,090 | € 15,900,247 | € 15,347,037 | € 14,685,049 |
| Adverse event management cost | € 11,344,632 | € 13,577,824 | € 16,255,546 | € 7,853,976 | € 7,580,716 | € 7,253,725 |
| **DPP4** | | | | | | |
| Overall cost | € 35,385,624 | € 33,607,342 | € 31,511,507 | € 32,033,980 | € 30,946,389 | € 29,686,897 |
| Pharmacological therapy cost | € 18,199,042 | € 17,284,461 | € 16,206,560 | € 16,475,271 | € 15,915,916 | € 15,268,152 |
| Complications management cost | € 15,851,152 | € 15,054,563 | € 14,115,724 | € 14,349,768 | € 13,862,577 | € 13,298,381 |
| Adverse event management cost | € 1,335,430 | € 1,268,319 | € 1,189,223 | € 1,208,941 | € 1,167,896 | € 1,120,364 |
| **Sulfonylureas** | | | | | | |
| Overall cost | € 30,330,577 | € 28,820,243 | € 27,040,430 | € 27,457,735 | € 26,525,513 | € 25,445,947 |
| Pharmacological therapy cost | € 3,928,002 | € 3,732,404 | € 3,501,907 | € 3,555,951 | € 3,435,222 | € 3,295,411 |
| Complications management cost | € 24,351,045 | € 23,138,466 | € 21,709,536 | € 22,044,571 | € 21,296,132 | € 20,429,397 |
| Adverse event management cost | € 2,051,530 | € 1,949,372 | € 1,828,988 | € 1,857,214 | € 1,794,159 | € 1,721,138 |
| **Metformin** | | | | | | |
| Overall cost | € 99,397,714 | € 94,448,128 | € 88,615,424 | € 89,982,994 | € 86,959,415 | € 83,421,447 |
| Pharmacological therapy cost | € 19,193,664 | € 18,237,901 | € 17,111,608 | € 17,375,685 | € 16,791,833 | € 16,108,653 |
| Complications management cost | € 73,972,044 | € 70,288,549 | € 65,947,835 | € 66,965,584 | € 64,715,429 | € 62,082,463 |
| Adverse event management cost | € 6,232,006 | € 5,921,678 | € 5,555,981 | € 5,641,725 | € 5,452,153 | € 5,230,331 |
| **All treatments** | | | | | | |
| Overall cost | € 301,677,535 | € 320,321,896 | € 342,847,243 | € 287,156,071 | € 287,314,832 | € 287,683,466 |
| Pharmacological therapy cost | € 143,572,673 | € 161,635,040 | € 183,335,321 | € 133,504,833 | € 134,759,839 | € 136,315,417 |
| Complications management cost | € 137,141,265 | € 135,969,663 | € 134,682,185 | € 135,013,030 | € 134,074,985 | € 133,067,317 |
| Adverse event management cost | € 20,963,597 | € 22,717,193 | € 24,829,738 | € 18,638,208 | € 18,480,008 | € 18,300,732 |
